# Characterization and functionality of *Ligilactobacillus agilis* 1003 isolated from chicken cecum against *Klebsiella pneumoniae*

**DOI:** 10.3389/fcimb.2024.1432422

**Published:** 2024-12-13

**Authors:** Hai chang Yin, Deng hao Jiang, Tian fei Yu, Xin jie Jiang, Di Liu

**Affiliations:** ^1^ College of Life Science and Agriculture Forestry, Qiqihar University, Qiqihar, China; ^2^ Postdoctoral Research Workstation, Heilongjiang Academy of Agricultural Sciences, Harbin, China

**Keywords:** *Ligilactobacillus agilis*, whole-genome sequencing, probiotic, antibacterial, metabolomics

## Abstract

Lactic acid bacteria are widely regarded as safe alternatives to antibiotics in livestock and poultry farming and have probiotic potential. *Ligilactobacillus agilis* (*L. agilis*) is a prominent component of pigeon crop microbiota; however, its function is unknown. In this study, a strain of *L. agilis* 1003 from pigeon cecum was identified by combining whole genome sequencing and phenotypic analysis, and its safety and probiotic properties were studied. Whole-genome sequencing revealed that the *L. agilis* 1003 genome length is 2.58 Mb, its average percent guanine-cytosine is 40.43%, and it encodes 1,757 protein-coding genes. Annotation of clusters of orthologous groups classified predicted proteins from the assembled genome as having cellular, metabolic, and information-related functions. A gene cluster associated with the synthesis of a broad-spectrum antimicrobial compound confirmed by antibacterial spectrum testing was identified using genome mining tools. Based on hemolysis test results, the strain was determined to be safe. This strain exhibited a high survival rate in the presence of bile salts and acidic conditions and a significant self-aggregation propensity and hydrophobicity. *In vivo* animal experiments showed that *L. agilis* 1003 exhibits probiotic and antibacterial effects and that the substances exerting antibacterial effects are organic acids. Metabolomics analysis revealed that *L. agilis* 1003 supernatant contained seven organic acids, including butyric acid. *L. agilis* 1003 showed good safety and probiotic potential in genomics, physiological biochemistry, and animal experiments, and could be considered a suitable candidate for promoting livestock and poultry health.

## Introduction

*Klebsiella pneumoniae* (*K. pneumoniae*) is a common gram-negative conditional pathogenic bacterium, a member of the genus *Klebsiella*, family *Enterobacteriaceae* (Jensen et al., 2020; Wang et al., 2018). It causes a wide variety of community-acquired infections in humans, including urinary tract infections, pneumonia, sepsis, and liver abscesses (Garza-Ramos et al., 2016; Rodriguez-Medina et al., 2019). *K. pneumoniae* has also been isolated from chickens with pharyngitis, dermatitis, cellulitis, and inflamed respiratory mucosa (Kowalczyk et al., 2022). According to a report from the US Centers for Disease Control and the European Centre for Disease Prevention and Control, resistance to broad-spectrum beta-lactam antibiotics by *K. pneumoniae* has become an alarming and growing public health challenge. The increasing incidence of multidrug-resistant bacteria poses a major challenge for physicians and veterinarians, limiting treatment options and raising concerns about transmission to humans through the food chain and the emergence of super-resistant bacteria (Li et al., 2022). Consequently, the potential risks of antibiotic use and the European Union’s prohibition policy on feeding antibiotics have underscored the benefits of using probiotics. This has, in turn, spurred the rapid development and application of probiotic feeding strategies (Ricke et al., 2020).

The widespread use of antibiotics has caused problems, including disruption of animal gut microbiota, the development of antibiotic resistance, and negative environmental impacts (Al-Khalaifa et al., 2019; Mulchandani et al., 2023; Ramirez et al., 2020). Many countries, including the European Union, the United States, and China, have enacted laws banning non-therapeutic antibiotic use in poultry and livestock farming (Ricke et al., 2020). Thus, there is an urgent need to identify effective antibiotic alternatives for use in livestock and poultry farming.

The production of broiler chickens faces challenges, including a high degree of intensification, high feeding density, and long-term accumulation of feces. The feeding cycle of broiler chickens is short, posing a great challenge to maintaining production levels after the prohibition of antibiotics. Dietary probiotics can change the intestinal structure by increasing the villi height in the duodenum and ileum of broilers and increasing levels of heat shock protein 70 (HSP70), thus alleviating the adverse effects of heat stress (Jiang et al., 2020). In broilers, *Ligilactobacillus salivarius* (*L. salivarius*) confers resistance and mitigates the negative effects of *Salmonella* infection. *L. salivarius* can also degrade Aflatoxin B1 and provide a protective effect to broiler chickens challenged with Aflatoxin B1, improving growth performance, liver function, and meat quality (Chen et al., 2022). In layer trials, *L. salivarius* CML352 was a suitable probiotic with positive effects on gut health and production performance (Xu et al., 2022). These scattered examples demonstrate that adding appropriate probiotics to maintain the balance of bacteria in poultry can alleviate diarrhea, boost immunity, enhance production performance, and mitigate other health problems.

Lactic acid bacteria (LAB) are the most studied and representative probiotics, and their metabolites, including bacteriocins and organic acids, represent good postbiotics (Mathur et al., 2020). In recent years, many studies have attributed highly diverse functions to probiotics, suggesting that their biological effects should be evaluated individually (Abdalla et al., 2021; Chen et al., 2022). Thus, it is necessary to conduct relevant analyses of newly isolated probiotics to explore potential biological functions and obtain information regarding their functional characteristics at the gene level (Saroj and Gupta, 2020; Tanizawa et al., 2015).

Although whole-genome sequences of many probiotic LAB have been reported, their whole-genome involvement and *in vivo* probiotic effects are still poorly understood (Goel et al., 2020; Qureshi et al., 2020). In our previous research, the probiotic strain *Ligilactobacillus agilis* (*L. agilis*) 1003 was isolated from the cecum of pigeons and proven to be a new probiotic that can produce active antibacterial substances. *L. agilis* is a prominent *Ligilactobacillus* component of the pigeon crop microbiota that has been only minimally studied (Baele et al., 2001). Therefore, the safety, probiotic capacity, and potential of this strain as an antibiotic alternative remain unknown. This study aimed to evaluate the safety and potential probiotic properties of *L. agilis* 1003 using a series of *in vitro* trials. In addition, whole genome sequence analyses were performed to provide greater insight into the full breadth of its biological capabilities and assess the safety and probiotic capabilities of *L. agilis*.

## Materials and methods

### Bacterial strains and growth conditions

The *L. agilis* 1003 strain was isolated from pigeon ceca, maintained at the Animal Immunology Laboratory of the College of Life Science and Agriculture Forestry, Qiqihar University, and cultured in Man Rogosa Sharpe (MRS) medium at 37°C in anaerobic jars for routine use. *Klebsiella* strains from chickens were cultured in Luria-Bertani (LB) medium at 37°C under aerobic conditions.

### Identification of *L. agilis* 1003

The morphology and phylogeny of *L. agilis* 1003 were evaluated as previously described (Fu et al., 2022). *L. agilis* 1003 was cultured on solid MRS Petri dishes at 37°C for 24 h, and colonies from the Petri dish were gram-stained. Their morphology was observed using scanning electron microscopy (SEM). Finally, the genotype of *L. agilis* 1003 was identified, and its *16S* rDNA sequence was analyzed and compared with the sequence deposited in the National Center for Biotechnology Information (NCBI) database using the Basic Local Alignment Search Tool (BLAST). A phylogenetic tree of *L. agilis* 1003 was constructed using the neighbor joining method in MEGA6 software.

### Whole genome sequencing, assembly, and annotation

Complete genome sequencing of *L. agilis* 1003 was performed by Shanghai Meiji Biomedical Technology Co. Ltd. (Shanghai, China). *L. agilis* 1003 cultured to the exponential stage was centrifuged at 8,000 × *g*, and total bacterial cell DNA was extracted using a DNA purification kit (Solarbio, Beijing, China). The whole genome was sequenced using the second-generation BGISEQ platform and third-generation PacBio sequencing technology. Subsequent genomic assembly was performed using Single-Molecule Real-Time sequencing technology (SMRT) Portal software package (v.2.3.0, Pacific Biosciences, Menlo Park, CA, USA) (Rhoads and Au, 2015).

The Prokaryotic Genome Annotation Pipeline (PGAP) algorithm (NCBI, Bethesda, MD, USA) was employed for *de novo* genome annotation (Tatusova et al., 2016). GeneMark software (v.4.17)1 was used to predict genome-wide protein-coding RNAs. Predicted sRNA, rRNA, and tRNA genes were identified using the search programs V1.1rc4, RNAmmer 1.2, and tRNAscan-SE V1.3.1 (Gabriel et al., 2024). Clustered Regularly Interspaced Short Palindromic Repeats (CRISPR) regions were identified using CRISPR digger V1.0, and plasmid information was obtained using the online plasmid Finder tool (Siguier et al., 2006). The Clusters of Orthologous Groups (COGs) of proteins and Kyoto Encyclopedia of Genes and Genomes (KEGG) databases were used for general functional annotation (Kanehisa et al., 2017). Genes related to toxin production and antibiotic resistance in the *L. agilis* 1003 genome were identified according to the antibiotic research database (CARD) (Alcock et al., 2020) and virulence factors database (VFDB) databases (Liu B. et al., 2022).

### Hemolytic activity analysis

Hemolytic activity assays were performed using a method described by Fu et al (Fu et al., 2022). Briefly, *L. agilis* 1003 and *Bacillus cereus* 2023ZXY cultures were crossed, streaked on Columbia blood agar containing fresh sheep blood, and incubated at 37°C for 24 h. Hemolytic activity was defined according to the following rules: If a colony (strain) produced a grass-green ring in the blood agar, it was considered to be α-hemolytic. If a colony produced a completely clear hemolytic ring in the blood agar, it was β-hemolytic. If no change was observed, the colony was considered non-hemolytic.

### Acid and bile salt resistance assessment

*L. agilis* 1003 was inoculated into 5 mL MRS liquid medium adjusted to different pH values (2.0, 3.0, and 4.0) and cultured at 37°C with shaking at 120 rpm for 24 h. Approximately 100 μL *L. agilis* 1003 solution was cultured on MRS agar medium at 37°C for 48 h, and bacterial growth on the plate was observed.

To assess bile salt tolerance, *L. agilis* 1003 was cultured at 37°C with shaking at 120 rpm in different bile salt concentrations (0.5, 1.0, and 1.5% [w/v]), and optical density (OD) values were measured at 630 nm. Bacteria from these cultures were spread on MRS agar, cultured at 37°C for 48 h, and examined for colony growth.

### Hydrophobicity test

Using phosphate buffered solution (PBS) as a blank control, the initial bacterial concentration was adjusted to yield an OD_600_ of approximately 0.6 ± 0.05 (A0). After adjusting the bacterial concentration, 4 mL of bacterial culture was added to 0.8 mL of xylene, vortexed at high speed for 2 min, and then allowed to stand for 10 min for phase separation. The lower water phase was removed, and light absorption was measured at OD_600_ (A1). Hydrophobicity (%) = (1−*A*1/*A*0) × 100

where A0 is the initial light absorption value of the bacteria, and A1 is the light absorption value of the bacterial solution mixed with xylene.

### Auto-aggregation capacity assay

Using PBS as a blank control, the initial bacterial concentration was adjusted to yield an OD_600_ of approximately 0.6 ± 0.05 (A0). The culture was placed at 37°C for 24 h, and culture medium OD_600_ was measured at 2, 4, 6, 12, and 24 h (A2). The auto-aggregation propensity of LAB cells was calculated using the following formula:


Self-coagulation rate (%)=(1−A2/A0)×100

where A0 is the initial light absorption value of the bacteria and A2 is the light absorption value of the culture medium.

### Evaluation of probiotic properties

#### Analysis of antimicrobial biosynthesis pathways in the genome

Antibacterial compound biosynthetic pathways were identified to clarify the antibacterial activities of *L. agilis* 1003. Biosynthetic gene clusters for each genome were annotated using AntiSMASH 6.0 with default parameters. Potential bacteriocin synthesis gene clusters were identified using the BAGEL4 web server.

#### Assessment of antimicrobial activity

*Klebsiella* strains from chickens were pre-cultured in LB liquid medium at 37°C for 12 h. The antibacterial activity of *L. agilis* 1003 cell-free supernatant against *Klebsiella* (10^7^ CFU/mL) was evaluated using the Oxford Cup double-plate method. Inhibition areas were measured using Vernier calipers, and sterile MRS broth was used as a control.

#### Broiler performance trial

We experimented with chickens to evaluate the probiotic functions of *L. agilis* 1003 *in vivo*. Forty-eight 14-day-old chickens were divided into four different groups of n = 12. They were fed 100 μL of *L. agilis* 1003 (10^7^ CFU/mL) or uninoculated MRS daily. Individual weights were recorded daily for 21 days, and then chickens were sacrificed by exsanguination via the jugular vein. Serum was isolated, and the cecal contents, spleen, bursa, and thymus were harvested and frozen at -80°C until use. Serum was used for cytokine analyses, cecal feces for intestinal microbiota analysis, and immune organs for immune organ index analysis. These experiments were approved by Qiqihar University and were conducted according to animal ethics guidelines and approved protocols

### Metabolite extraction and preparation

*L. agilis* 1003 was incubated anaerobically in MRS medium at 37°C for 72 h and conditioned media was obtained by centrifugation (6,000 rpm, 10 min) followed by filtration through a 0.22 µm sterile membrane. The liquid was completely frozen in liquid nitrogen for three freeze-thaw cycles, and then intracellular metabolites were extracted and collected by centrifugation (12,000 × *g*, 20 min, 4°C). A volume of 120 mL supernatant was carefully transferred into 2 mL injection bottles, and 10 mL of each sample was mixed into quality control samples for Ultra Performance Liquid Chromatography Tandem Mass Spectrometry (UPLC-MS) analysis using Vanquish UHPLC system (Thermo Fisher, Bremen, Germany) with Orbitrap Q Exactive HF-X Mass Spectrometer (Thermo Fisher).

### Metabolome data analysis

Compound Discoverer (v.3.1; Thermo Fisher Scientific, Waltham, MA, USA) was used to process the original data generated by UHPLC-MS/MS, and peak comparison, peak selection, and quantification of each metabolite were performed. Peak intensity was normalized to the total spectral intensity. The normalized data predicted molecular formulas based on additive ions, molecular ion peaks, and fragment ions. The peaks were matched to the mzCloud, mzVault, and Masslist databases to obtain accurate qualitative and quantitative results. These metabolites were identified using the KEGG database (https://www.genome.jp/kegg/pathway.html).

### Statistical analysis

All experiments were conducted in triplicate, and each sample was analyzed in triplicate. GraphPad Prism software (v.9.0) was used for all statistical analyses. Error bars represent the standard error of the mean in all figures, and *P*-values were determined using unpaired two-tailed Student’s *t*-test.

## Results

### Isolation and identification of *L. agilis* 1003

**Figure 1:**
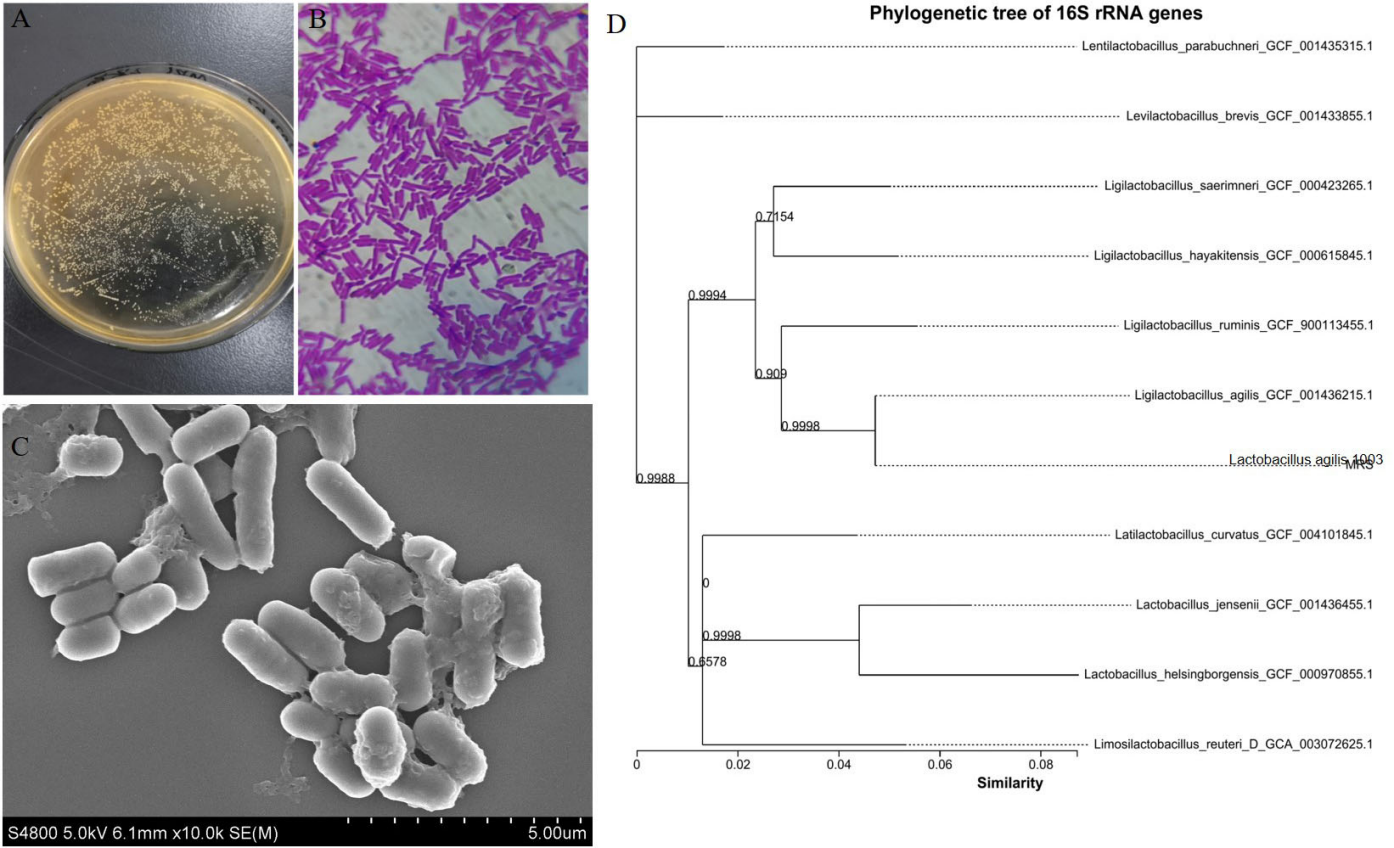
Morphological characteristics and phylogenetic tree of *L. agilis* 1003. **(A)** Colony morphology; **(B)** *L. agilis* 1003 gram staining; **(C)** SEM observations; and **(D)** phylogenetic tree. All sequences are from *Lactobacillus* strains. Other *Lactobacillus* species were used as outgroups. Numbers at nodes indicate bootstrap values of neighbor-joining analyses with 1,000 replicates.

Morphological observations revealed that *L. agilis* 1003 colonies were round, white, flat, rough, wet, and formed neat edges (**Figure 1A**). *L. agilis* 1003 bacteria are gram-positive and exist as single long rods (**Figure 1B**). SEM confirmed that the cells were rod-shaped, and no spores or flagella were observed (**Figure 1C**). A phylogenetic tree constructed using the neighbor connection method in Mega6 software revealed similarity between the *L. agilis* 1003 strain and *Ligilactobacillus*_*agilis*_GCF_001436215.1 to be ≥ 99% based on *16S* rDNA analysis (**Figure 1D**).

### Genomic characterization of *L. agilis* 1003

**Figure 2:**
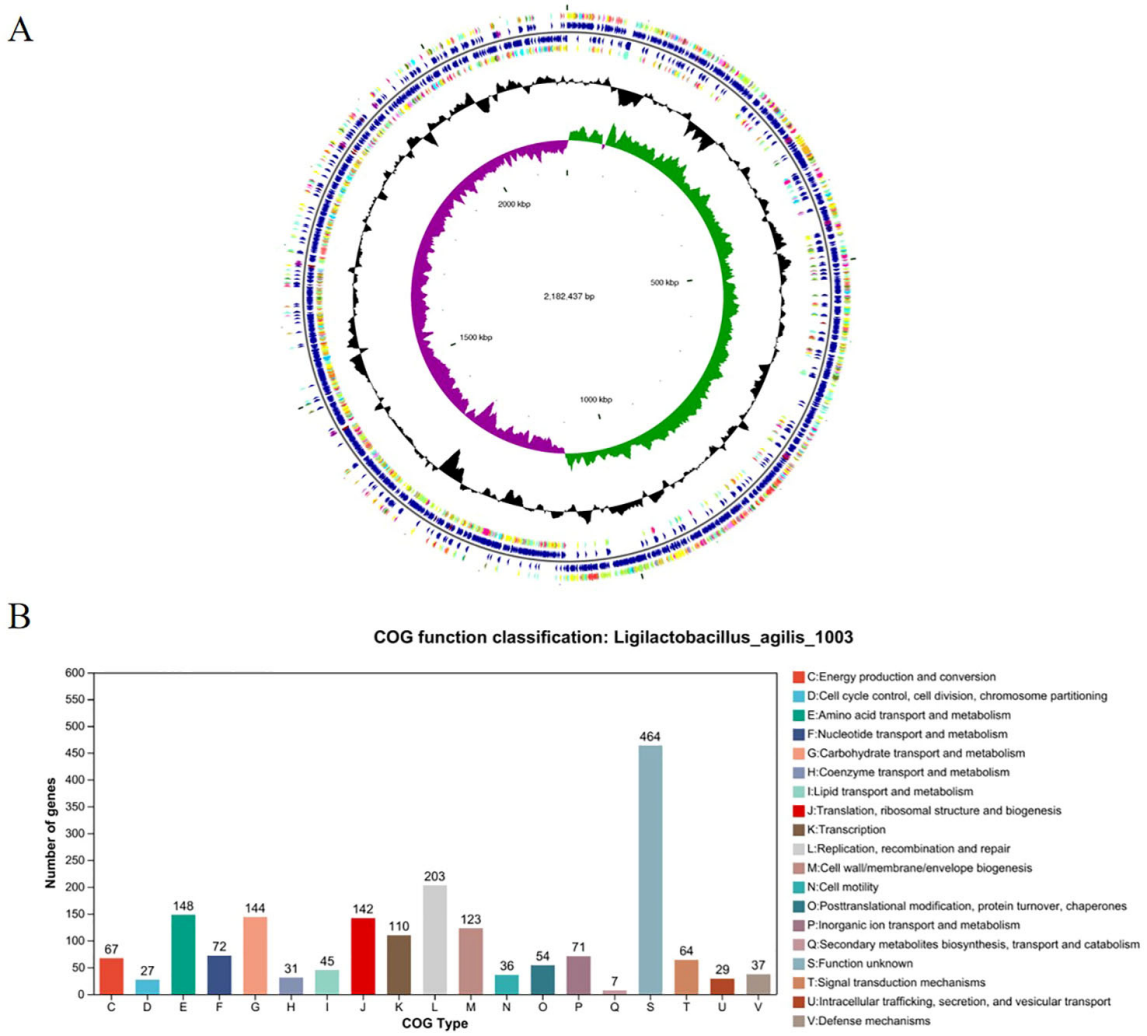
Genomic characterization and analysis of *L. agilis* 1003. **(A)** Circular genome map of *L. agilis* 1003; **(B)** Distribution of genes across COG functional categories in the *L. agilis* 1003 genome.

Whole-genome sequencing of *L. agilis* 1003 revealed a genome size of 2.58 Mb with a single circular chromosome and a GC content of 40.43%. Three circular plasmids were identified and designated: plasmid 1 (158,146 bp), plasmid 2 (234,098 bp), and plasmid 3 (4,518 bp) (**Figure 2A**). A total of 2,468 protein-coding gene sequences, 96 tRNA genes, 24 rRNA genes, and 39 sRNA genes were identified (**Table 1**). The 1,846 protein-coding genes were assigned to families comprising 19 functional classes divided into four categories: cellular, metabolic, informational, and genome assembly. COG classification results showed that unknown function, amino acid transport and metabolism, carbohydrate transport and metabolism, translation, ribosomal structure and biogenesis, replication, recombination, and repair-related proteins accounted for large respective proportions (23.94, 7.64, 7.43, 7.33, and 10.47%) (**Figure 2B**).

**Table 1:** General features of the *L. agilis* 1003 genome.

Attribute	Value
Genome size (bp)	2579199
GC content(%)	40.43%
Plasmid	3
Total RNA	159
Number of rRNAs	24
Number of tRNAs	96
Number of sRNAs	39
Number of protein-coding genes	2468
Number of prophage region	3

Genes related to toxin production and antibiotic resistance in the *L. agilis* 1003 genome were identified according to the Comprehensive Antibiotic Research Database (CARD) and virulence factors database (VFDB) databases, respectively. A total of 83 putative virulence factor genes were identified based on the VFDB database. The similarity of only 12 putative virulence factor genes with VFDB was > 60%, while < 74%. Based on CARD, 110 genes associated with antibiotic resistance were identified, and only two resistance genes with > 60% similarity were covered, including rifamycin (rpoB2) and streptogramin (vatI) resistance-related genes. In addition, the three plasmids identified did not contain any resistance genes. The results obtained from ResFinder and DisinFinder indicated that no resistance genes were detected, confirming the compliance of *L. agilis* 1003 with the essential safety criteria for probiotic use.

### Bile salt and acid tolerance

The effects of different acid and bile salt concentrations on *L. agilis* 1003 are shown in **Table 2**. At bile salt concentrations of 0.5, 1.0%, and 1.5%, the respective survival rates of *L. agilis* 1003 were significantly reduced to 81.90%, 44.59%, and 23.08%, compared to those in the control group. When cultured at pH 2, 3, or 4 for 5 h, the respective *L. agilis* 1003 survival rates decreased to 57.91% (*P* < 0.05), 83.91% (*P* < 0.05), and 92.24% of control culture survival.

**Table 2:** *L. agilis* 1003 bile salts and pH tolerance.

Treatment	Time(h)	Viable count (×10^7^CFU/mL)	Survival rate (%)
Control	5	7.02 ± 0.37	100
0.5% bile salt	5	5.75 ± 0.19	81.90
1.0% bile salt	5	3.13 ± 0.36	44.59
1.5% bile salt	5	1.62 ± 0.13	23.08
pH 2	5	4.12 ± 0.21	57.22
pH 3	5	5.98 ± 0.12	85.19
pH 4	5	6.67 ± 0.34	95.01

### Self-aggregation and hydrophobicity

We observed an *L. agilis* 1003 aggregation rate of 49.23 ± 1.09% and a hydrophobic index of 63.27 ± 1.23%.

### Hemolytic activity analysis of *L. agilis* 1003

**Figure 3:**
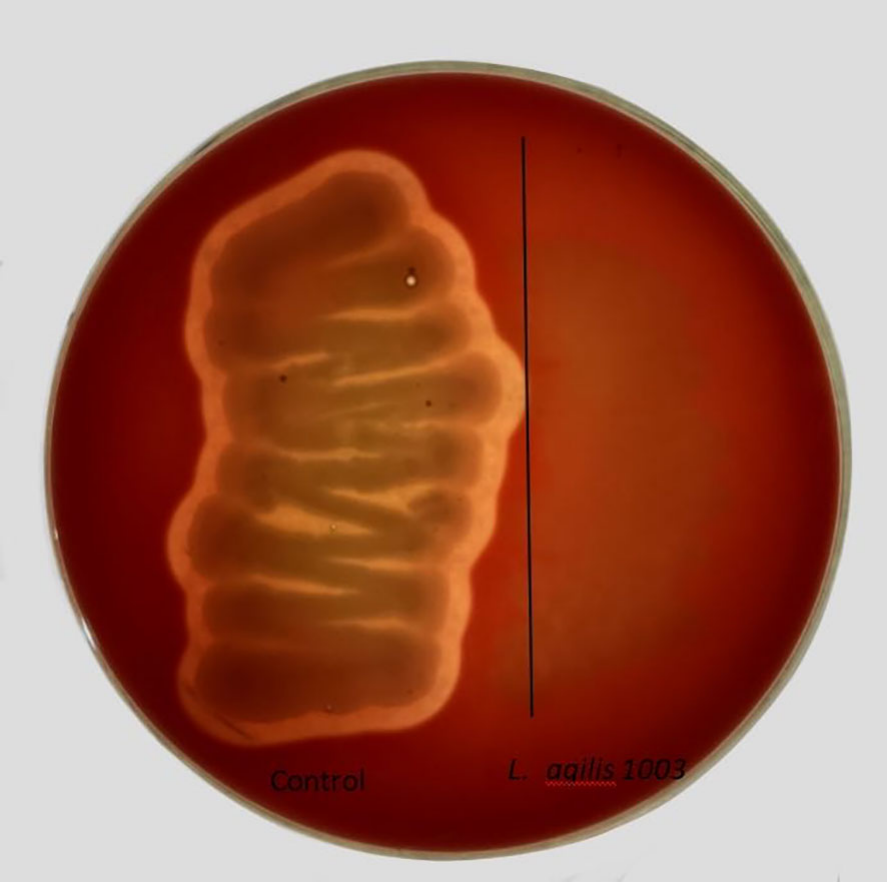
Assessment of *L. agilis* 1003 hemolytic capability. The positive control *Bacillus cereus* 2023ZXY produced an obvious zone of β-hemolysis (left); *L. agilis* 1003 showed no hemolytic activity (right).

Many bacteria produce hemolysins that can hemolyze blood cells on agar plates. This capability results in a hemolytic ring around colonies of such bacteria. A control bacterium isolated from chicken cecum, *Bacillus cereus* 2023ZXY, was observed to be β-hemolytic, whereas *L. agilis* 1003 displayed no hemolytic activity (**Figure 3**).

### Evaluation of *L. agilis* 1003 probiotic properties

**Figure 4:**
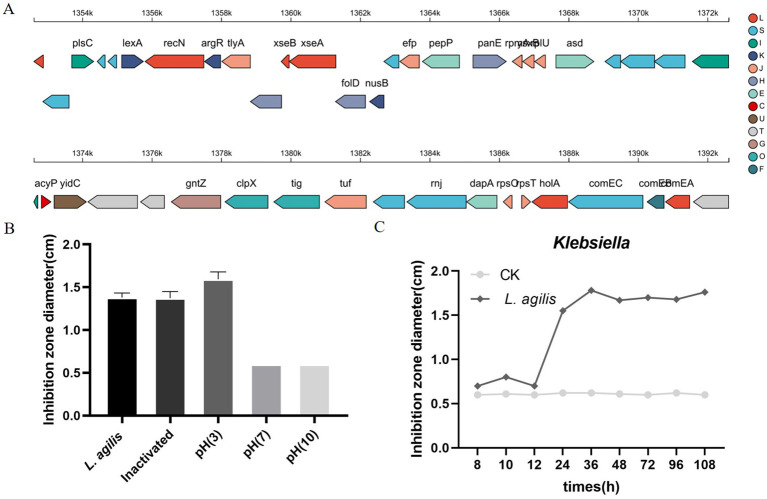
Evaluation of *L. agilis* 1003 probiotic properties and activity against pathogens. **(A)** Gene clusters encoding T3PKS are represented by arrows, with different colors corresponding to operons encoding different functions; **(B)** Diameters of inhibition zones of conditioned media from isolated strains under different pH and heat treatments were measured and are represented as means ± SD of 3 independent replicates; **(C)** Diameters of inhibition zones of conditioned media obtained from isolated strains after different cultivation times were measured and are represented as means ± SD of 3 independent replicates. Wells containing 50 mg/mL gentamycin were positive controls, and wells containing sterile MRS medium were negative controls.

AntiSMASH 5.0 and BAGEL 4.0 databases were used to identify possible antimicrobial genes in the *L. agilis* 1003 genome to evaluate encoded probiotic capabilities. AntiSMASH identified one gene associated with type III polyketide synthase (T3PKS), whereas BAGEL4 did not identify any areas of interest (**Figure 4A**). *L. agilis* 1003-conditioned media produced an inhibitory ring diameter of 18 mm against *Klebsiella* compared to that in the control group. Thermal treatment did not affect this antibacterial activity. However, the antibacterial activity disappeared when the pH was adjusted to neutral or alkaline (**Figure 4B**). The antibacterial effect of the supernatant was most potent after culturing *L. agilis* 1003 for 72 h (**Figure 4C**). It is noteworthy that hydrochloric acid did not inhibit the growth of *Klebsiella*, indicating that the antibacterial activity is independent of pH in conditioned media and instead relies on specific metabolites. These data suggest that the anti-*Klebsiella* effects can be attributed to organic acids in the metabolites of *L. agilis* 1003 rather than proteins.

### Profiles of metabolites

**Figure 5:**
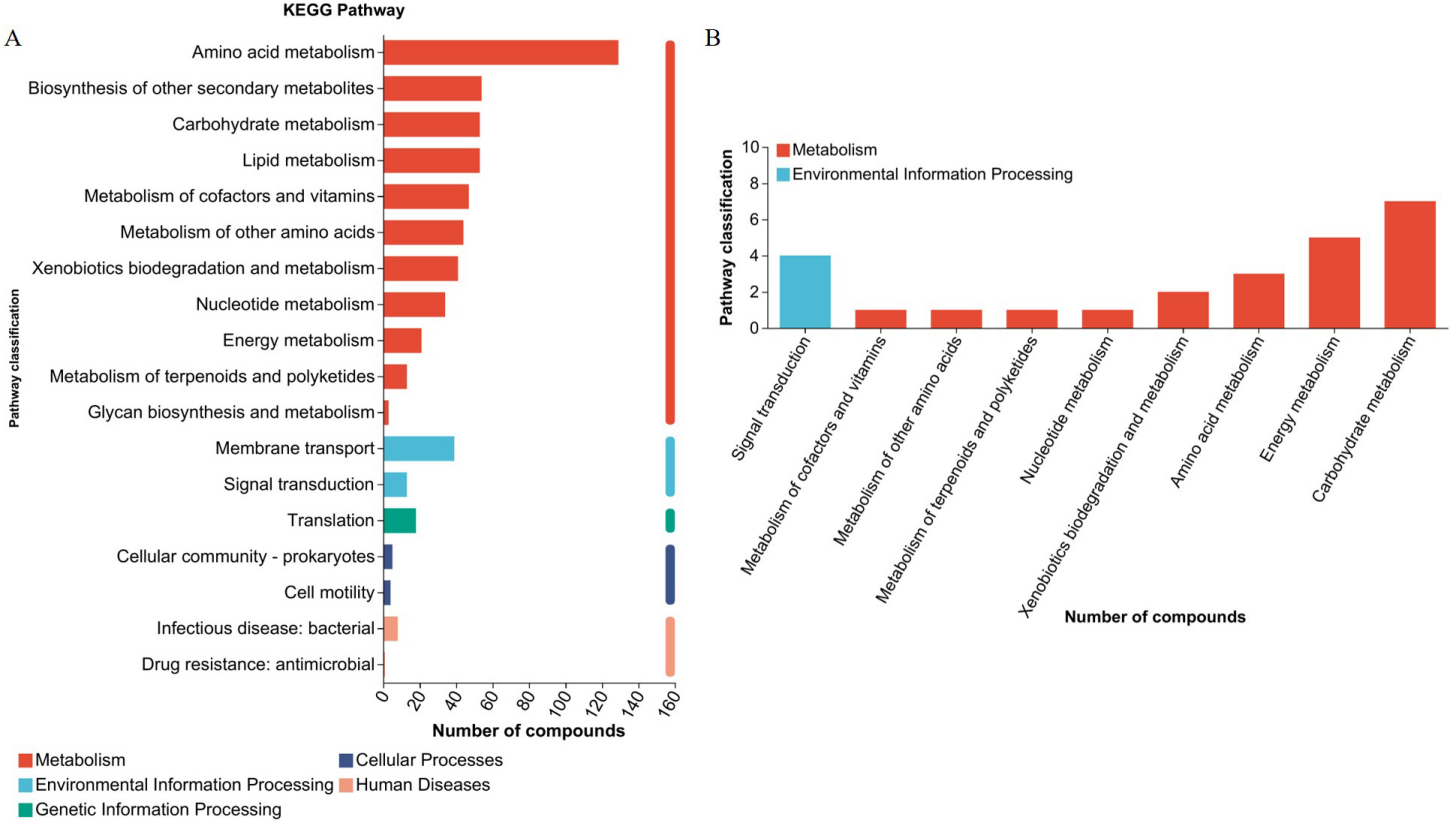
*L. agilis* 1003 conditioned media metabolite profiles. **(A)** KEGG annotation pathways for all metabolites; **(B)** KEGG annotation pathways for organic acids.

UHPLC-MS/MS analysis detected 1,915 metabolites after filtering the internal standard and false positive peaks. Among these metabolites, 890 were detected in positive mode and 1,015 in negative mode. Functional annotations were obtained using the KEGG metabolome database to investigate biological correlations of the identified metabolites. The main enriched functional pathways identified by KEGG in the metabolic class were amino acid metabolism, biosynthesis of other secondary metabolites, carbohydrate metabolism, and lipid metabolism. The main identified membrane transport class pathway was translation in genetic information processing, and the main environmental information processing class pathway was translation (**Figure 5A**). Seven organic acid components were identified, including succinic acid, trans-acetic acid, acetoacetic acid, hydroxypropionic acid, citric acid, isocitric acid, and malic acid. According to KEGG enrichment analysis, the main metabolic class-enriched pathways were carbohydrate metabolism, energy metabolism, and amino acid metabolism. The main environmental information processing class pathway was signal transduction. No annotations associated with any other first category were identified (**Figure 5B**). Whether organic acids is the main components underlying *L. agilis* 1003 antibacterial activity or not, and their precise mechanisms of action require further investigation.

### Enhances early broiler performance

**Figure 6:**
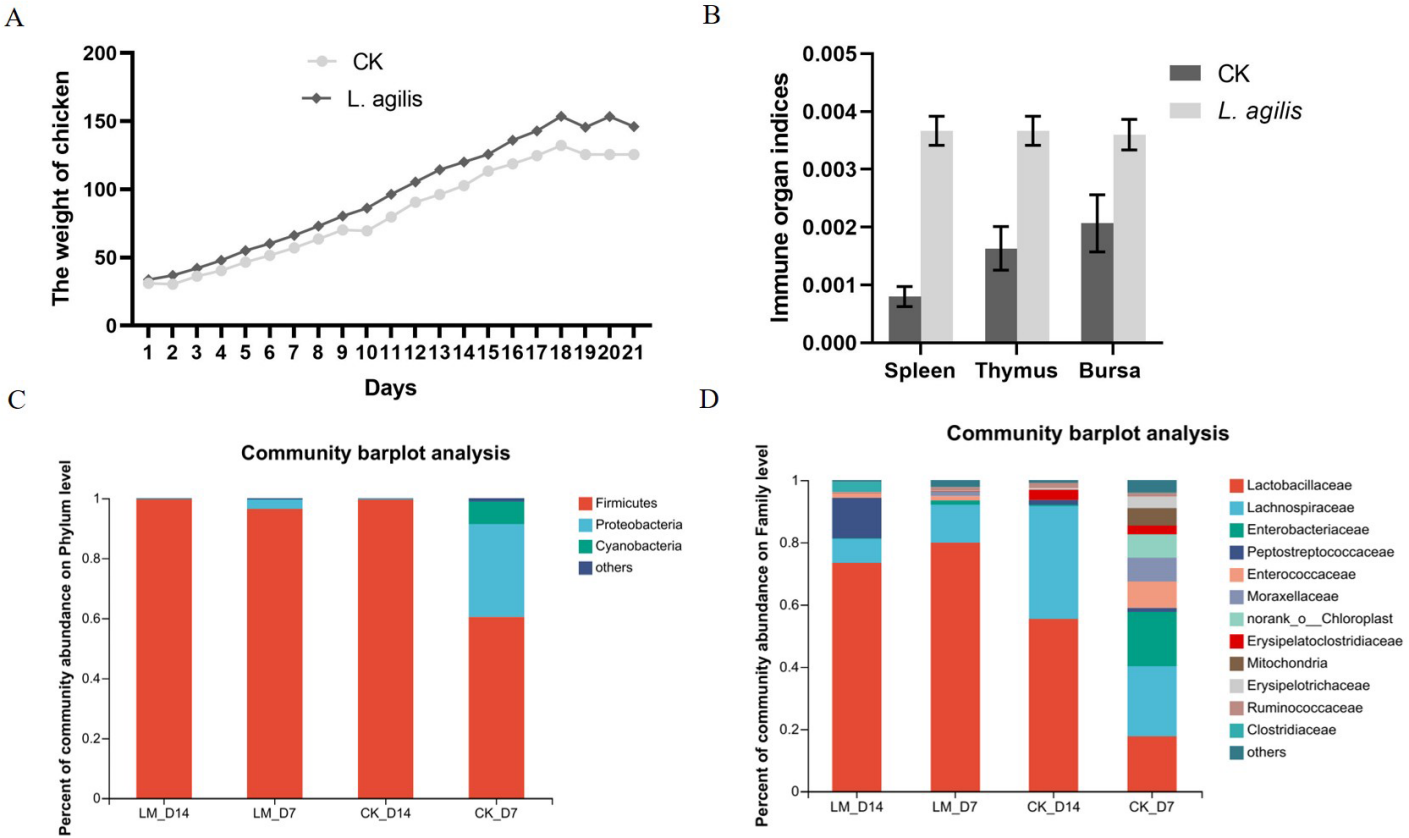
Evaluation of *L. agilis* 1003 probiotic effect in broilers. **(A)** Broiler weight changes over 21 days; **(B)** Immune organ indices of the spleen, bursa, and thymus; *16S* DNA sequencing of changes in intestinal microbial abundance in broilers fed *L. agilis* 1003 at Phylum **(C)** and Family **(D)** levels. LM_D7 indicates feeding *L. agilis* 1003 bacterial for 7 days; LM_D14 indicates feeding *L. agilis* 1003 bacterial for 14 days. CK_D7 and CK_D14 represent controls, respectively. Data are shown as means ± SD from three independent experiments, **P < 0.01. Statistical variance analysis was calculated using Student’s *t*-test.

The average body weight of broilers at 21 days of age in the *L. agilis* 1003 group was higher than that in the control group. However, this difference was not statistically significant (*P* > 0.01) (**Figure 6A**). Similarly, immune organ indices of the spleen, thymus, and fabricius bursa were significantly increased in broilers inoculated with *L. agilis* 1003 (**Figure 6B**). In addition, the *Lactobacillus* proportion of Firmicutes in the intestinal microbiota of broilers fed *L. agilis* 1003 was significantly increased (**Figures 6C, D**), indicating that *L. agilis* 1003 exerts a significant probiotic effect *in vivo*.

## Discussion

Probiotics are active microorganisms that benefit the host by changing the composition of the flora in certain parts of the body through colonization, which can reduce the incidence of diarrhea and promote the absorption of nutrients (Al-Khalaifa et al., 2019; Zheng et al., 2021). Providing appropriate probiotics to livestock and poultry to maintain a beneficial composition of bacteria in the host body can alleviate health problems, including diarrhea, decreased immunity, and decreased production performance (Zheng et al., 2021). However, because the efficacy of probiotics depends on the species or strains present, commercial probiotics should meet a specific set of characteristics for safety, functionality, and benefits (Li et al., 2020). In this study, we elucidated the safety and potential probiotic properties of *L. agilis* 1003 through a series of *in vitro* and *in vivo* trials combined with whole genome sequencing and metabolomics. We analyzed antibacterial genes and compounds to reveal their potential biological functions.

The development of new strain resources and the evaluation of strain safety are necessary to obtain the most effective bacteria (Ye et al., 2020). In this study, *L. agilis* 1003 exhibited no hemolytic activity, indicating that the *L. agilis* 1003 strain is non-toxic. In addition, dietary supplementation with *L. agilis* 1003 induced no adverse effects on the growth performance or overall health of broilers. These results indicate that *L. agilis* 1003 is safe for livestock and poultry breeding.

Tolerance to bile salts and acidic conditions are key characteristics for candidate probiotics because the presence of bile salts and highly acidic conditions represent the greatest barriers to survival for *Lactobacilli* in the gastrointestinal tracts of animal hosts (Toropov et al., 2020; Ye et al., 2020). In this study, after 5 h of treatment with 1.5% bile salts and 5 h of exposure to pH 2, the survival rates of *L. agilis* 1003 were 23.08% and 57.22%, respectively, demonstrating higher tolerance than by other probiotic LAB strains (Xu et al., 2023; Zheng et al., 2021). Self-agglutination and hydrophobicity are important characteristics for efficient probiotic colonization of animal intestines (Goel et al., 2020; Qureshi et al., 2020). In this study, *L. agilis* 1003 self-aggregation and hydrophobicity values were 49.23% and 63.27%, respectively, indicating its potential ability to survive intestinal and gastric environments and adhere to intestinal epithelial tissue.

Growth performance is the most direct index of broiler production, and optimization is important to improve economic benefits. Adding LAB to feed has been reported to improve animal growth performance. Shokryazdan et al. (Shokryazdan et al., 2017) reported that adding an *L. salivarius* mixture to the diet significantly increased the average daily gain and feed ratio of cobalt broilers. Peng et al. (Peng et al., 2016) added *Ligilactobacillus plantarum* B1 to the diet and observed a sharp increase in daily gain and a significant increase in feed coefficient. Humam et al. (Humam et al., 2019) reported that the final weight, total weight gain, and average daily weight gain of Cobb broilers supplemented with *Ligilactobacillus plantarum* were significantly higher than those of a control group. In this study, the average body weight of broilers at 14 days in the *L. agilis* 1003 group was higher than that in the control group. However, this difference was not statistically significant (*P* > 0.01). The observed increase in body weight may be attributable to the ability of probiotics to regulate digestive enzyme activity, improve intestinal digestion and absorption of nutrients, and promote animal growth (Jager et al., 2018), indirectly suggesting that *L. agilis* 1003 does not promote fat accumulation in broilers.

Immune organs (thymus, spleen, bursae, etc.) are the main tissues in poultry that generate specific immunity. Their developmental status directly affects the ability of poultry to resist invasion by pathogens and is an important index for evaluating the growth and immune functions of poultry (Wang et al., 2017). Chen et al. (Chen et al., 2022) showed that *Lactobacillus* KL1 and *L. plantarum* combined with probiotics could effectively improve the immune organ index of broilers. In this study, feeding *L. agilis* 1003 significantly improved the immune organ indices of broilers. The content of the immune factor IFN-β in the blood of chickens in the treatment group was higher than that in the control group. In addition, *L. agilis* 1003 supplementation significantly increased intestinal *Ligilactobacillus* content in broilers and regulated their intestinal microecological balance. These results indicate that *L. agilis* 1003 plays a beneficial role in broilers.

Probiotics can competitively inhibit pathogen adhesion to intestinal mucosal epithelial cells, compete with pathogens for limited nutrients and biological targets, and prevent pathogen growth and reproduction (Abd El-Hack et al., 2020). *L. agilis* 1003 conditioned media has demonstrated high antibacterial activity against the pathogenic bacterium *Klebsiella* in the intestinal tract of chickens and pigeons and may act via organic acids, consistent with many studies (Liu G. et al., 2022; Mani-Lopez et al., 2022; Wang et al., 2023).

Due to the advantages of whole genome sequencing and the diversity of genome mining tools, it is possible to predict the ability of strains to produce antimicrobial compounds (Fu et al., 2022). In this study, The AntiSMASH 6.0 prediction results identified a gene presumed to be associated with the T3PKS antimicrobial compound in the *L. agilis* 1003 genome. The antibacterial effect may be due to organic acid metabolites, and metabolomics analysis of *L. agilis* 1003 supernatant revealed seven classes of organic acids. Which of these organic acids plays a role in *L. agilis* 1003 antibacterial effects merits further exploration.

## Data Availability

Raw DNA sequence reads were deposited with the NCBI (accession number CP156820). All data are available upon request from the authors.
